# Evaluation of the impact of a 3-week specific-sport rehabilitation program on neuromotor control during single-leg countermovement-jump tests in professional soccer players with lower-limb injuries

**DOI:** 10.3389/fspor.2024.1448401

**Published:** 2024-12-05

**Authors:** Geoffrey Memain, Christopher Carling, Jean Bouvet, Pascal Maille, Bertrand Tamalet, Paul Fourcade, Eric Yiou

**Affiliations:** ^1^ FIFA Clairefontaine Medical Center, French Football Federation, Clairefontaine-en-Yvelines, France; ^2^ CIAMS Laboratory, Université Paris-Saclay, Orsay, France; ^3^ CIAMS Laboratory, Université D'Orléans, Orléans, France; ^4^ French Football Federation Research Centre, Clairefontaine National Football Centre, Clairefontaine-en-Yvelines, France; ^5^ Laboratory Sport, Expertise and Performance (EA 7370), French Institute of Sport (INSEP), Paris, France

**Keywords:** neuromotor control, rehabilitation, elite soccer, CMJ, lower-limb injuries, LSI, norm values

## Abstract

**Purpose:**

This study investigated the evolution of neuromotor control during a typical short sport-specific rehabilitation program (SSR) in professional soccer players who had incurred a major lower-limb injury (*n* = 15, chondral and muscle injuries, ACL-reconstruction).

**Methods:**

All injured participants (*n* = 15) were in the on-field rehabilitation phase of their specific sport rehabilitation process, prior to return to play. An experimental group (EG, chondral and muscle injuries, ACL-reconstruction) followed a 3-week SSR-program composed of muscular and core strengthening (weightlifting, functional stability, explosivity and mobility exercises), running and cycling, neuromotor reprogramming, cognitive development and specific soccer on-field rehabilitation (acceleration, braking, cutting, dual-contact, high-speed-running, sprint, jump, drills with ball). Neuromotor control via analysis of movement kinematics, muscle activation and kinetic parameters was evaluated using a single-leg Countermovement-Jump, pre- and post- rehabilitation program. A control group (*n* = 22) of healthy soccer players of similar standards performed the same single-leg Countermovement-Jump to provide reference values regarding the level to be attained by the injured players for return to play.

**Results:**

In the experimental group, almost all kinetic analyses values progressed during the program and significantly for concentric Rate-of-Force-Development (*p* < 0.05), height jump (*p* < 0.001) and Reactive-Strength-Index Modified (*p* < 0.001) but remained lower than control group values for RSI-Mod (*p* < 0.05) and RFDconcentricLate (*p* < 0.001). Activation changed (*p* < 0.05) for all muscles except for rectus femoris and medial gastrocnemius in the pushing phase and rectus femoris during landing in the EG. Activation of all muscles decreased for EG, except for semitendinous which increased. Regarding kinematic analyses during the landing phase, there were a significant decrease in peak trunk flexion (*p* < 0.001) and lateroflexion (*p* < 0.001) and an increase in peak knee flexion (*p* < 0.001) for both legs. Trunk flexion (*p* < 0.001) and lateroflexion (*p* < 0.001) values were again higher for EG while knee flexion remained significantly lower than the CG (*p* < 0.001).

**Conclusion:**

The SSR generally improved neuromotor control suggesting that the present specific sport rehabilitation program, albeit of only three weeks duration, was effective in aiding elite footballers recover their neuromotor qualities although this was potentially insufficient to return to the values observed in healthy players.

**Level of evidence:** Therapeutic studies of level II.

## Introduction

Contemporary elite soccer is imposing ever-increasing levels of stress on players notably due to increases in the frequency of matches and competitive physical demands (1). While over the last 20-years the incidence of joint and ligament injuries has decreased, hamstring and ligament injury severity has concomitantly increased (2, 3). Injury recurrence rates are high, attaining values of 36% for knee chondral injuries (CH), 17.5% for lower-limb muscle injuries (MI) and 6.6%–10% for anterior cruciate ligament ruptures (4) (ACL). These injuries generate various alterations including arthrogenic muscular inhibition (AMI) (5), pain interference (6), detraining or sensorimotor impairments (7). These can have a strong impact on lower-limb functioning. Indeed, RFD deficits ranging from 10% to 57% have been reported up to 24 months after ACL reconstruction in the injured and non-injured leg (8, 9). For thigh muscle strains, a weakness in eccentric force production has been observed, notably in external muscle range of motion post hamstring injury (10). Moreover, a chondral injury generates inappropriate activation and muscle imbalances leading to impaired dynamic coordination (11).

As such, sport-specific rehabilitation programs (SSR) (12–15) aim to ensure complete restoration of any affected functions and safe and efficient return-to-play phases (RTP) (16). SSR generally include neuromotor training and reprogramming (17–19) (strengthening, postural-work, core-training, mobility, motor-learning, locomotor exercises), physiological energy system conditioning, cognitive work (17) and specific on-field rehabilitation exercises. Buckthorpe et al. (17) suggest that on-field rehabilitation is constructed around 4 pillars: fitness, movement quality, sport-specific skills, and training load. The sport-specific rehabilitation phase can be organized in 5 distinct phases where the intensity, volume, complexity and specificity of the exercises and sessions on the field are progressively increased. One example is the “control-chaos continuum” (CCC) proposed by Taberner (14). The athlete must be able to perform all the movements occurring in their sport (cutting, shifting, jumping, landing, shooting, contact, sprinting, braking, acceleration, processing information and decision-making), all at maximum intensity, repeatedly over time, and with quality movement (18, 20).

To support decision-making during RTP, medical and reconditioning staff frequently utilize information derived from motor evaluations (17, 21, 22) (e.g., hop, landing, isokinetic, agility tests) commonly performed in clinical settings. However, these tests might not be considered discriminating enough to specifically assess any motor deficits that might persist in injured athletes (23) during RTP. As such, RTP assessment batteries frequently including multiple tests have been proposed (22). However, it is not always practically or logistically easy to perform several tests. One test, the Countermovement-Jump (CMJ) is useful as a performance measure (24), a means to evaluate neuromotor control deficits, and also a readiness to play measure (24, 25) while limiting core and limb compensations.

During RTP processes, the between-leg (a)symmetry derived from analyses of neuromotor control is commonly investigated using a “leg symmetry index” (LSI, percentage difference in values for a selected variable between both legs) when performing a locomotor task (20, 26). The LSI-method is used to evaluate neuromotor control impairment and recovery (21, 27) in injured athletes performing lower-limb tests. Recovery is generally considered “complete” if LSI = 100% (20, 26). However, it has been suggested that the LSI overestimates players’ progress in returning to play (28, 29). The utilization of “normative” values is relevant where comparisons of the athlete's current post-injury state can be made with reference values that are both reliable and level-appropriate in cohorts of healthy players (14, 30, 31).

The purpose of this study was to assess the effects of a typical 3-week SSR-program on neuromotor control recovery in the injured and non-injured legs of elite soccer players. Analyses of kinematic, kinetic and EMG (electromyography) variables derived from a single-leg CMJ (SLCMJ) *before* and *after* the SSR-program would help determine the variables most impacted during the players' rehabilitation over the 3-week period. The hypothesis forwarded is that the SSR would have a positive impact on all variables, thanks to its comprehensive, functional, and systematic approach whilst bringing the results in both legs of injured players closer to reference values observed in a control group of healthy players.

## Materials and methods

### Experimental approach to the problem

The effect of a 3-week SSR-program (13–15) on neuromotor control was investigated in injured male professional soccer players. Two groups were formed: an experimental (EG, *n* = 15) comprised of players with a unilateral lower-limb injury and a control group (CG, *n* = 22) comprised of uninjured (healthy) players of the same playing standard. All players performed 3-unilateral CMJ using each leg; before and after the SSR-program for the EG and during a single-session for the CG. Metrics included whole-body kinematics, kinetics, and lower-limb muscle activation.

### Participants

The cohort included an EG composed of players having sustained a lower-limb injury (Chondropathy *n* = 4, Muscle Injury *n* = 4, Anterior Cruciate Ligament-rupture *n* = 7) and receiving treatment at the Clairefontaine FIFA Medical Center of the French Football Federation, and a CG. The CG (*n* = 22) included players who had not incurred any significant injury (absence longer than one week) during the six months before the study. All injured participants were in the advanced part of their rehabilitation, the final “on-field rehabilitation” phase, the aim of which, irrespective of the injury, is to regain the ability to meet the demands of competitive practice in all areas. The groups presented similar anthropometric characteristics (Table 1). This study complied with the Declaration of Helsinki (1964) and permission was obtained from French national ethics committee for sports science research (CERSTAPS n°IRB00012476-2020-24-03-48).

**Table 1:** Comparison of anthropometric characteristics between EG and CG.

	Size			Mass			Age		
	CG	Pre-SSR	Post-SSR	CG	Pre-SSR	Post-SSR	CG	Pre-SSR	Post-SSR
Subjects	22	15	15	22	15	15	22	15	15
Mean	181.8	180.7	180.7	75.1	76.6	76.8	24.8	26.6	26.6
Std. deviation	7.1	4.9	4.9	7.2	8.8	8.8	3.6	4.4	4.4
Minimum	168.0	173.0	173.0	60.0	61.5	63.0	20.0	19.0	19.0
Maximum	192.0	189.0	189.0	87.0	89.0	89.0	32.0	33.0	33.0

### Experimental task and protocol

The experiments were performed between 2:00 and 3:30 PM in a training-room, at 20°C. The protocol began with a 10-min warm-up on an ergocycle followed by a progressive increase in power from 100W to 200W. Two maximal isometric voluntary contractions (MVIC) of the leg muscles were then performed for EMG normalization, followed by series of unmeasured SLCMJ trials (three per leg) on a force-plate. These blank trials were performed to ensure familiarization with the experimental task, apparatus, and instructions. Following a one-minute rest period, the two series of SLCMJ were repeated and recorded.

Participants performed barefoot with their hands fixed on their hips. They started in a static position with their stance leg stretched and the contralateral leg slightly flexed with the foot a few centimeters above the force-plate. Participants returned to the same posture following SLCMJ. They were instructed to “jump as high and as quickly as possible and stabilize themselves three-seconds in the final posture”.

Following this first series of tests (pre-SSR), EG participants followed the SSR-program over a 3-week period, which corresponds to a micro-cycle work unit duration within a typical rehabilitation program (13, 14). It also corresponds to the average duration of an injured player's stay at the present Football Medical Center. Post-program, the EG repeated the testing protocol (post-SSR). CG only performed a single test as conducting the same tests twice in healthy top-level footballers is difficult notably due to logistics regarding their training and competition schedules in addition to the effect of the associated loading. In addition, some of the players were no longer able to perform the test battery as they no longer met the inclusion criteria on being injury free. A pilot study in the twenty-two CG subjects showed that the raw experimental variables did not differ significantly when SLCMJ was performed on their dominant or non-dominant leg (*p* > 0.05). As such, only the dominant leg was tested in the CG.

### SSR-program

The SSR-program (13, 14, 32) was composed of muscular strengthening (weightlifting and functional exercises), physiological energy system conditioning running and cycling, neuromotor reprogramming and specific soccer on-field rehabilitation (acceleration, braking, cutting, dual-contact, high-speed-running, sprint, jump, drills with ball) on the pitch, core-training, mobility and cognitive development (2, 20, 32–36). EG players performed the program approximately 5-h per day, 5-days a week, during 3-weeks consecutively. On each day of the on pitch SSR-program, players performed mobility, specific lower-limb activation, neuromotor control and specific soccer rehabilitation in the morning and lower-limb strengthening, core-training and specific care in the afternoon. The soccer-specific rehabilitation part included general and specific drills and soccer movement (accelerations, decelerations, cutting, jump, landing, dribble, shift), high-speed-running and sprinting, short and long passes, duels and physical contact work, and cognitive work (information analysis and decision making). A progressive augmentation in the volume, intensity and complexity of the content of the pitch sessions was implemented over the 3weeks following previous recommendations for on-field rehabilitation (12, 20, 32–34, 36, 37). The external and internal workloads and intensities were monitored and adapted in relation to progression, according to the characteristics of the injury and the individual's response to the programme. This was done to respond as effectively as possible to the inter-individual differences in adaptation and recovery times. The exercises were ceased if the player deemed the pain was greater than 3/10 using a numerical rating scale (38). The decision criteria for changing training focus depended upon the progress made during strength training assessments and GPS tracking data. The program was monitored by a certified physical trainer specialized in rehabilitation and a team of sport physiotherapists working under the responsibility of three medical doctors specialized in sport rehabilitation. A weekly planning of the SSR program is available in the appendixes.

### Data recordings

**Figure 1:**
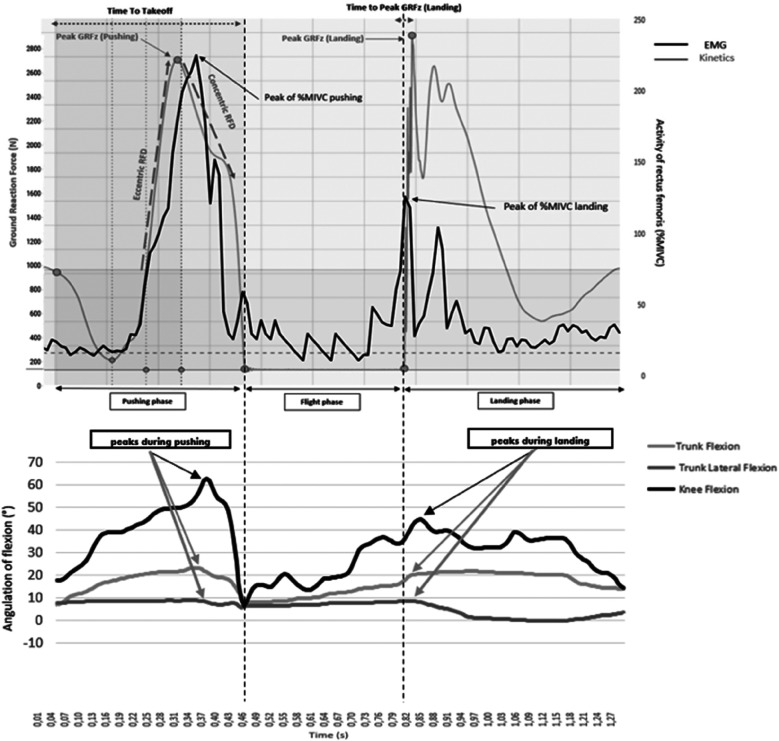
Example of electromyogram, kinematics and kinetics curve of a control group athlete during a single-leg countermovement jump on a strength platform. For the EMG and kinetic curves, a drop occurs in the curve corresponding to the countermovement (the unloading of athlete's weight), then the curves rise during the athlete's reloading phase (end of the eccentric) to the top of the curves. The curves then drop again (concentric thrust phase), then there is a slight plateau (with zero values for the kinetic) corresponding to the flight time, then finally the curves rise again during the jump landing phase before plateauing again.

SLCMJ movement was analyzed though kinetic, kinematic and EMG data recording (Figure 1). Kinetic data was obtained using a force-plate (9260AA6 Kistler Instruments, Hampshire, UK) that provided ground reaction force (GRF_Z_), and moments applied at its surface. Kinematic data for knee flexion (KF), trunk flexion (TF) and lateroflexion (TLF) of the stance leg was obtained using the Humantrak system (Vald Performance, Brisbane, Australia) with a Kinect-v2 camera (Microsoft Corp., Redmond, WA, USA). Kinematic positional data was processed through a dual Butterworth filter to remove residual noise. Electrical activity of lower-limb muscles was recorded with 12-channel Delsys Trigno (39) wireless surface Ag/AgCl sensors (27 mm × 35 mm, Trinoma, Lyon, France): vastus medialis (VM), rectus femoris (RF), biceps femoris (BF), semitendinous (ST), gluteus medius (GM) and medial gastrocnemius (MG). SENIAM (40) recommendations were applied for sensors location. EMG signals were filtered by a 10-Hz bandpass filter (41) and by a Butterworth filter in EMGworks 4.4 software (Delsys, Inc.) via Root Mean Square. All recordings were sampled at 1,000 Hz.

### EMG normalization

The electrical activity of each muscle obtained during propulsion and landing phases of SLCMJ series was normalized with respect to maximal voluntary isometric contraction (MVIC) (41). EMG activity during MVIC was evaluated by two successive MVIC for 5-s separated by a 30-s rest interval for each muscle studied in both legs, before each test session. The highest maximal averaged value obtained on the sliding 0.5-s periods (the highest average recorded over a period of 0.5 during the 5-s isometric contraction) was considered MVIC (41). This was assessed during specific analytical exercises carried out on a guide machine, with the targeted muscle contracting against an over-maximal resistance. MVIC was evaluated with the leg extended at 45° knee flexion for the vastus medialis and rectus femoris and for the leg curl at 45° knee flexion for the biceps femoris and semitendinous using a fixed pulley at 25° of abduction for the gluteus medius and on the calf press with the leg extended and neutral ankle position for the medial gastrocnemius (40).

### Raw experimental variables

The following spatio-temporal and kinetics variables were obtained from the force-plate (Figure 1):

- Peaks of upward vertical ground reaction force (peak vGRF, in *Newton*) produced during the pushing phase and during the landing phase of the single-leg-CMJ (24). The landing phase corresponds to the dynamic phase after the flight phase.
- Jump height (in cm) represents the maximal altitude attained by the athlete during the single-leg-CMJ, estimated by the force-plate software through flight duration. Values are a functional measure of the athlete's neuromotor performance (24).
- The Reactive Strength Index Modified (RSI-mod, in m/s) is the ratio of jump height to time to take-off (countermovement duration). This metric reflects lower-limb explosiveness (24).
- Rate-of-Force Development (RFD, in N/s) during both the eccentric (RFDeccentric) and concentric (RFDconcentric) phases of the pushing motion (see Figure 1). RFDeccentric is determined by the slope of the line between the return to the athlete's body weight while ascending the ground reaction force (GRF) and the first upward peak of the vertical GRF trace. RFDconcentric is defined as the slope of the line from this first upward peak to take-off time (23). These parameters reflect lower-limb explosiveness.
- The vertical ground reaction force value at *t* = 50 ms after foot landing (vGRF at 50 ms landing, in Newtons). This moment is known to coincide with the peak risk of knee injury (42, 43).
- Time to peak vertical ground reaction force during the landing phase (in ms), indicating the duration between foot landing and the peak of the vertical ground reaction force.

The EMG parameters encompassed both the peak and mean values of electrical activity in the leg muscles, expressed as a percentage of the activity observed during maximal isometric voluntary contraction, throughout both phases of the single-leg-CMJ (see Figure 1).

Kinematic variables included peak knee flexion, peak trunk flexion, and peak trunk lateroflexion angles (in degrees) recorded during both phases of the single-leg-CMJ (see Figure 1) (7, 44).

### Statistics

Group means and standard deviations were computed for VAR_IL_, VAR_NIL_ and VAR_CT_ raw variables in pre- and post-SSR. The Shapiro-Wilk test was used to check the normality of the data distribution. To assess the neuromotor capacity of IL and NIL, repeated measures (RM) ANOVAs included the method (3-levels: VAR_IL_, VAR_NIL_ vs. VAR_CT_) and SSR (2 levels: pre-SSR vs. post-SSR) as within subject factors were used on each VAR_IL_, VAR_NIL_ and VAR_CT_. A significant outcome was followed by the Tukey *post hoc* test to assess pairwise statistical differences between methods and both SSR conditions. A student *T*-test was used to compare anthropometric data between the two experimental groups. Kinematic values remain expressed in degrees (°) as values can be positive or negative, so percentage methods were not relevant in this context. The significance threshold was set at *p* < 0.05. Cohen's d was used to determine the effect sizes for differences in mean values (classified as trivial: <0.2, small: 0.2–0.49, medium: 0.5–0.79, and large: ≥0.8).

## Results

### Comparison of anthropometric characteristics between groups

No significant differences were observed in inter-group characteristics (*p* > 0.05).

### Impact of the SSR-program on IL and NIL according to the estimation method chosen

#### Kinetic analysis

Results reported no differences for all RFD_eccentric_ and peak vGRF pushing phase values (*p* > 0.05) for both legs. In contrast, a significant impact of the SSR was observed for RFD_concentric_ (*F* = 7.8, *p* < 0.01), RFD_concentricEarly_ (*F* = 4.4, *p* < 0.05), RFD_concentricLate_ (*F* = 5.9, *p* < 0.05), RSI-Mod (*F* = 12.4, *p* < 0.001), jump height (*F* = 13.6, *p* < 0.001), time to peak of vGRF landing (*F* = 4.0, *p* < 0.05) and vGRF at 50 ms of landing (*F* = 8.7, *p* < 0.01). *Post hoc* tests revealed that the SSR-program increased RSI-Mod in IL (*p* < 0.01, *d* = 1.1), jump height in IL (*p* < 0.001, *d* = 0.8) and vGRF at 50 ms during landing in NIL (*p* < 0.05, *d* = 0.6).

**Figure 2:**
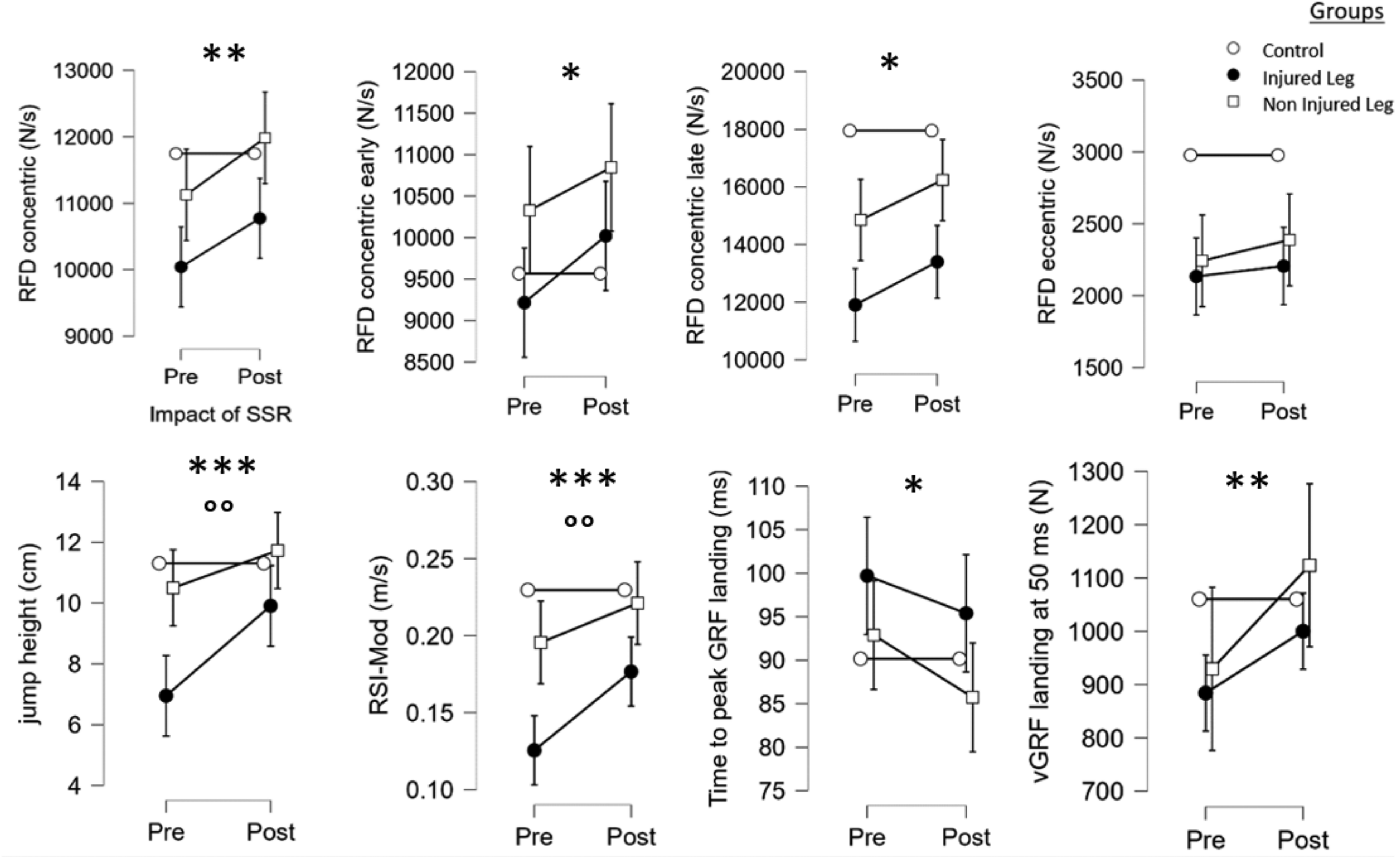
Main effects of the SSR program and the group on the kinetics variables of the single-leg-CMJ. *^,^ **^,^ ***: significant effect of the SSR program (pre vs. post-SSR) with *p* < 0.05, *p* < 0.01 and *p* < 0.001, respectively. °^,^ °°^,^°°°: significant effect of the group with *p* < 0.05, *p* < 0.01 and *p* < 0.001, respectively.

There were a significant group*SSR interaction between IL, NIL and CT for jump height (*F* = 5.4, *p* < 0.01) and RSI-Mod (*F* = 4.4, *p* < 0.05). *Post hoc* tests revealed that post-SSR, there was no significant difference between IL, NIL and CT, except for the RSI-Mod and RFD_concentricLate_ where IL was lower than CT (*p* < 0.05, *d* = 0.6 and *p* < 0.001, *d* = 0.8), underlining progression in both legs towards the values observed in the healthy players (see Figure 2).

#### EMG analysis

Results showed that the SSR-program led to significant differences for%MIVC mean of semitendinous (*F* = 15.9, *p* < 0.001), %MIVC max of vastus medialis (*F* = 6.0, *p* < 0.05), rectus femoris (*F* = 5.7, *p* < 0.05) and gluteus medius (*F* = 7.6, *p* < 0.01) muscles during pushing. Some significant differences were observed for%MIVC mean of biceps femoris (*F* = 6.3, *p* < 0.05), medial gastrocnemius (*F* = 7.6, *p* < 0.01) and for%MIVC max of vastus medialis (*F* = 6.6, *p* < 0.05), biceps femoris (*F* = 5.8, *p* < 0.05), semitendinous (*F* = 23.6, *p* < 0.001), gluteus medius (*F* = 11.2, *p* < 0.001) and medial gastrocnemius (*F* = 9.5, *p* < 0.01) muscles during landing. *Post hoc* tests revealed that the SSR-program led to an increase in pushing phase mean%MIVC for the semitendinous in the IL (*p* < 0.05, *d* = 0.5) and NIL (*p* < 0.01, *d* = 0.6), in%MIVC max for the semitendinous in the IL (*p* < 0.01, *d* = 0.7) during landing. Decreased max%MIVC of the gluteus medius for the NIL (*p* < 0.01, *d* = 0.6) and of medial gastrocnemius for NIL (*p* < 0.001, *d* = 0.7) were observed during landing.

**Figure 3:**
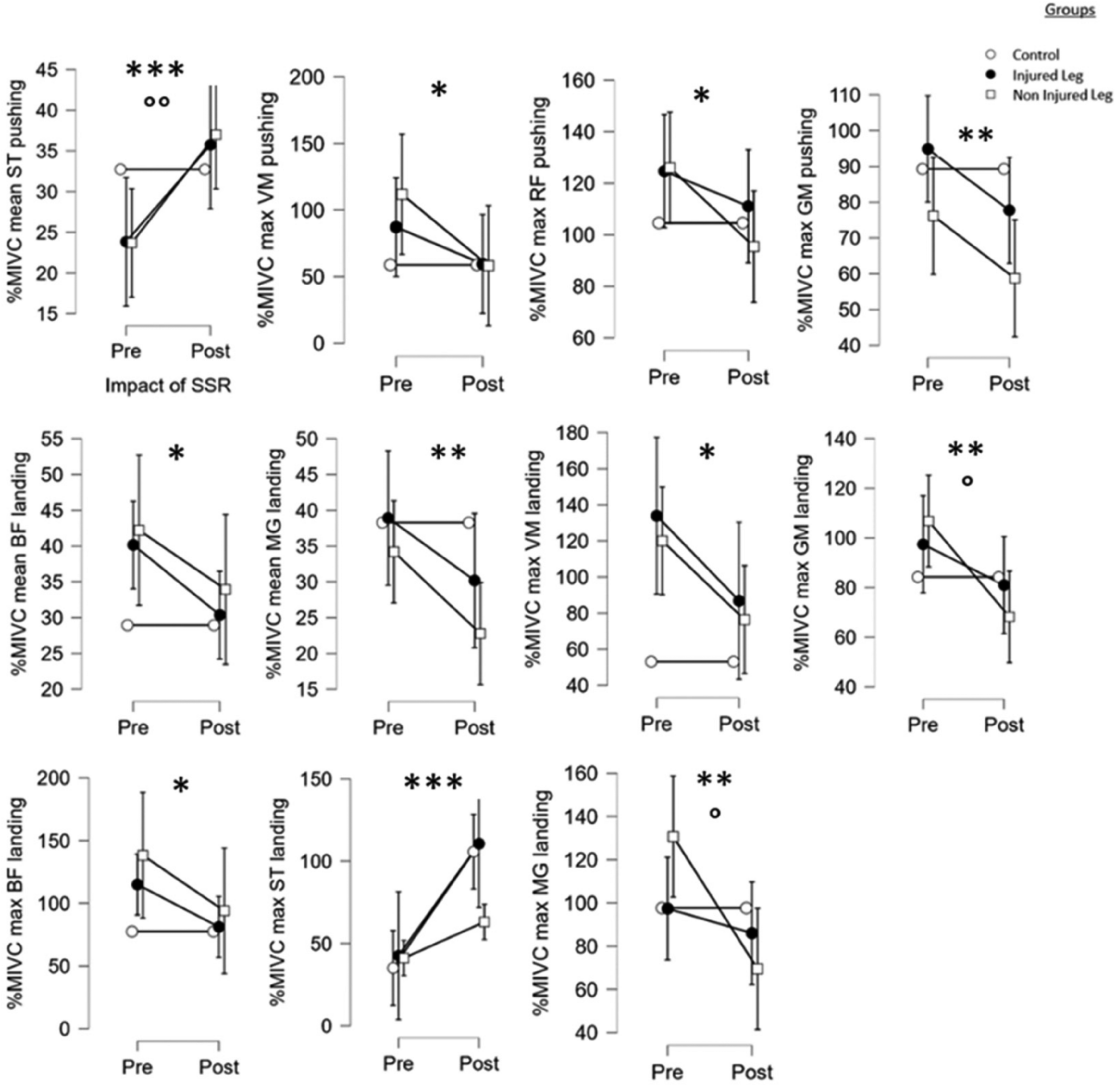
Main effects of the SSR program and the group on the EMG variables of the single-leg-CMJ. *^,^ **^,^ ***: significant effect of the SSR program (pre vs. post-SSR) with *p* < 0.05, *p* < 0.01 and *p* < 0.001, respectively. °^,^ °°^,^ °°°: significant effect of the group with *p* < 0.05, *p* < 0.01 and *p* < 0.001, respectively.

There was a significant group*SSR interaction for%MIVC mean of semitendinous during pushing (*F* = 4.5, *p* < 0.01), for%MIVC max of gluteus medius (*F* = 4.4, *p* < 0.05) and medial gastrocnemius (*F* = 5.5, *p* < 0.05) during landing (see Figure 3).

#### Kinematic analysis

Results showed that SSR-program led to significant differences in active knee flexion at push (*F* = 5,4, *p* < 0.05,), at landing (*F* = 26,4, *p* < 0.001); and in trunk flexion (*F* = 50,7, *p* < 0.001) and trunk lateroflexion (*F* = 405,9, *p* < 0.001) during the landing phase. *Post hoc* tests revealed that the SSR-program led to a decrease in knee flexion of NIL at push (*p* < 0.001, *d* = 0.5), a knee flexion of NIL increase at landing (*p* < 0.001, *d* = 1.0) and a decrease in IL and NIL values, respectively for trunk flexion (*p* < 0.001, *d* = 3.2 and *p* < 0.001, *d* = 2.5) and trunk lateroflexion of IL (*p* < 0.001, *d* = 1.1) during the landing phase.

**Figure 4:**
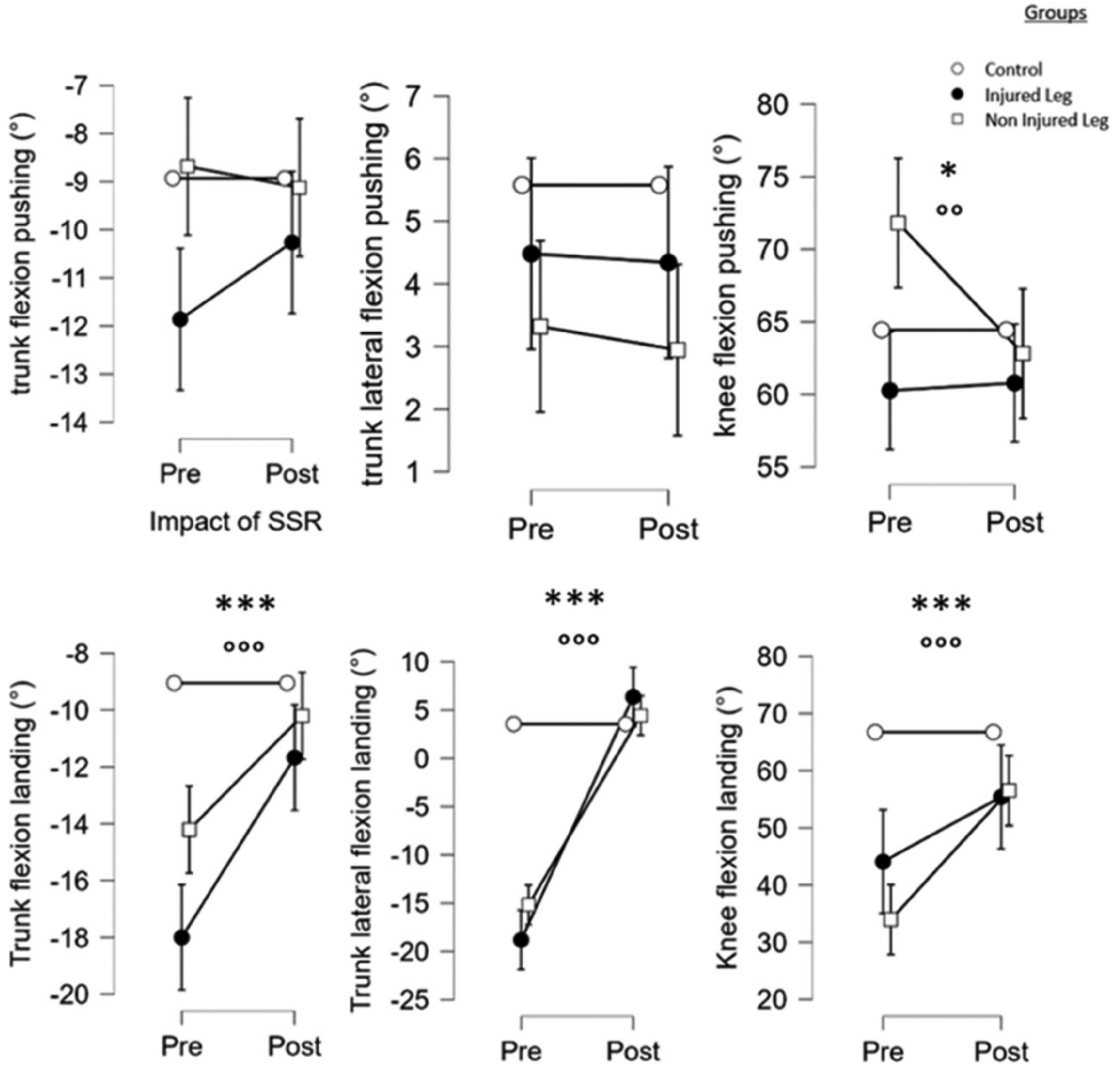
Main effects of the SSR program and the group on the kinematics variables of the single-leg-CMJ. *^,^ **^,^ ***: significant effect of the SSR program (pre vs. post-SSR) with *p* < 0.05, *p* < 0.01 and *p* < 0.001, respectively. °^,^ °°^,^ °°°: significant effect of the group with *p* < 0.05, *p* < 0.01 and *p* < 0.001, respectively.

Finally, there were a significant group*SSR interaction between IL, NIL and CT for knee flexion (*F* = 9.5, *p* < 0.001), trunk flexion (*F* = 16.3, *p* < 0.001) and trunk lateral flexion (*F* = 123.3, *p* < 0.001) during landing (see Figure 4).

## Discussion

### Impact of the SSR-program

The present study firstly assessed the effectiveness of a typical 3-week SSR-program on neuromotor control for the injured leg (IL) in a group of professional soccer players. The SSR-program positively impacted several neuromotor parameters for both the IL and non-injured leg (NIL). Similar results have previously been observed related to the impact of neuromuscular training but with an eccentric dominance during muscle-strengthening program (35). Here, strength conditioning and neuromotor control work coupled with high-intensity and soccer-specific movements generated significant gains in RFD, despite the program's short duration (3-weeks). Improvement in neural aspects in the RFD_Early_ phase including muscle activation, reduction of recruitment threshold and increase in the rate of motor-unit discharge or a facilitation of spinal and supraspinal outputs can potentially explain this gain (8, 9, 45–47). Structural contractile factors are preponderant for the RFD_Late_ phase (8, 9, 45–47). These include muscle fiber architecture, composition, and strength level. Concentric gains are achieved more quickly because the coordination, muscle recruitment and neural adaptations specific to this contraction regime are less complex (35). In contrast, no progress was observed in the injured players for RFD_EccentricLate_. To our knowledge, this variable has not yet received any attention in the literature. Nevertheless, we can hypothesize that a 3-week period is potentially insufficient to establish nerve force adaptations unlike structural ones (21, 35). Other possible explanations include a lack of quality eccentric explosiveness based on muscular pre-activation and pre-synaptic facilitation (48, 49); or self-confidence to descend quickly during a post-injury ballistic movement. As such, players must continue neuromotor training to improve inter and intramuscular coordination and optimize this eccentric-concentric transition period (48–50).

Regarding muscle activation, the semitendinous was the muscle most positively impacted by the SSR, with a significant increase observed. In relation to the associated literature, one would expect a significant increase in muscle activation following neuromotor training (45, 51, 52). This is linked to improvements in corticomotor excitability, motor-unit synchronization and a reduction in inhibitory processes, and even after only 1-week of targeted work (52). To generate the same force, an eccentric contraction requires less muscle activation than a concentric one (53). An increase in discharge frequency and recruitment of slow motor-units to distribute the mechanical stress applied to the muscle can be responsible for stimulating EMG activity (53). These observations were observed only for the semitendinous and rectus femoris (not significatively) in IL and may partly be explained by the general rehabilitation program performed pre-SSR (11–13, 19). Indeed, this enabled the players to resume running and perform one-leg jumps without pain as nerve adaptations related to activation had certainly already been stimulated. Hamstrings and quadriceps are preferential targets of central and peripheral inhibitions (AMI) (5) and pain adaptation (6) potentially explaining this evolution. Research has previously discussed the compensation capacity of the BF when ST is inhibited (54). Here, SSR enabled a higher activation of the semitendinous avoiding overactivation of the BF and can therefore be partly responsible for any stagnation. RF and MG did not change with this result possibly explained by the quadriceps being targeted at an early stage during rehabilitation in order to eliminate any possible inhibition (5), and the athletes use better coordination reducing the compensations by the ankle and therefore the MG. Research, albeit limited (55), has also shown a lack of change in muscle activation after a neuromotor program and no correlation between jump height and muscle activity (20).

Regarding kinematic data, analysis of SLCMJ demonstrated noteworthy results. IL and NIL exhibited higher trunk flexion during pushing and landing probably to compensate for a lack of knee flexion, but these were less important post-SSR. When excessive trunk lateroflexion is observed, a deficit in pelvic control with a gluteus medius deficit in functionality is highlighted (56). These improvements show that the SSR-program had a positive impact on kinematic compensations linked to the injuries and particularly improved the neuromotor function of both the IL and NIL. This is possibly due to progress in activation qualities, muscle strength and confidence in one's leg movement. Indeed, poor neuromotor control of the trunk impacts dynamic knee stability, resulting in increased abduction and tension on the ligaments and joint (16, 56). The present SSR-program increased knee flexion at landing to dampen the movement, and knee flexion at push were very similar for IL, NIL and CG after SSR, demonstrating the effectiveness of the SSR-program effects on knee functionality. Knee flexion, knee valgus and hip abduction have previously been studied (41, 48) and reported the same observations as here (16, 56). The reduction in TLF may be due to a better utilization of knee flexion (linked to activation, strength and RFD), limiting distal instability and therefore compensation with the trunk. This improvement in trunk control is essential as postural stabilization deficit of the trunk is a key risk factor for joint and muscle injury (16, 56). Increasing knee flexion and trunk flexion during jump and landing actions would reduce GRF_Z_ levels and therefore the stress on body structures to limit injury risk (56, 57). Dynamic postural deficits have been observed up to 9–12 months following ACL-reconstruction highlighting that there are still postural compensations that have not yet been normalized during rehabilitation, as reported in our results (8, 9). As such, the SSR-program seems to be efficient for ensuring intrinsic functionality recovery of the knee. The kinematic analyses underline compensations, orientate rehabilitation and prophylactic work, and evaluate players' capacity to return to play using qualitative control of movements (14, 56–58). Different characteristics related to neuromotor control were observed depending on the injuries studied. These are currently under investigation in a sister paper (entitled, Modeling the neuromotor capacities of professional soccer player with a lower-limb injury during a Countermovement Jump, submitted).

A recent review on return-to-play deplores that this process is too often based on subjective data and lacks objectivity, normalization, standardization and scientific consensus (59). To validate any RTP process, specialized literature including the Italian Consensus Conference recommend either no LSI deficit or values less than 10% and return-to-performance levels prior to injury (9, 14, 60–62). Our pre-SSR result of deficits in RFD_concentric_ (32%), jump height (8%) and RSI-Mod (17%) highlights that the NIL is also impacted (9) by injury to the contralateral leg and therefore also requires specific care and reconditioning during rehabilitation. This deconditioning of the NIL is consistent with the rate of reinjury reported in the contralateral leg over the two years following an initial ACLR injury (63). Moreover, if the NIL has not undergone specific conditioning training, RFD deficits of LSI (10%–57%) have been reported up to 24 months after surgery (8, 9) in the IL and NIL, which is outside the acceptability threshold for any RTP process. Once again comparison with healthy players seems to be of prime importance in guiding the rehabilitation staff on the program to be implemented for injured players. However, when this NIL training is performed, NIL values are closer to healthy players' performance, as our results showed with a reduction observed for deficits in RFD_concentric_, jump height and RSI-Mod respectively of 24%, 0% and 4% also underlining the efficacity of the SSR-program on NIL.

This study has two main limitations. First, the cohort size which can be explained by limited access to elite standard participants. Secondly, the GE was made up of players with different types of injury (ACLR, muscle strain and chondral injury) who have different rehabilitation durations and severities of neuromuscular alteration, which may complicate analysis of the impact of the present SSR. Three weeks is a “normal” duration for the SSR of muscle strains, whereas according to the literature, ACL and CH require a longer period of SSR.

## Conclusion

The main objective of any RTP process is to monitor and aid the athlete's return from injury and ultimately help them respond to the specific demands of competition (14, 55). SLCMJ tests during the RTP process are pertinent, useful and discriminating in the evaluation of neuromotor control (14). A SSR-program, even of 3-weeks duration, was effective in recovering neuromotor qualities in a group of high-level footballers although progress in their return to play programme seemed insufficient to attain the same level as healthy players. Moreover, results also showed a positive impact of the SSR-program on the players' NIL, underlying that it is essential to develop the capacities of the both the IL and NIL, to help avoid deficits on return to play with the NIL. Monitoring the progress of athletes during a SSR is important to refine the program and RTP strategies to minimize the risk of injury recurrence (20, 22, 64). Despite this, pending a scientific and clinical consensus on the objectification of SRR and the RTP processes, the impact of sport-specific rehabilitation program arguably depends mainly on the quality of the physical trainer in charge (59).

## Data Availability

The raw data supporting the conclusions of this article will be made available by the authors, without undue reservation.
